# Global Status of Research on Lateral Lymph Nodes in Rectal Cancer from 1994 to 2022: A Bibliometric Analysis

**DOI:** 10.3390/healthcare11101362

**Published:** 2023-05-09

**Authors:** Yang Zhang, Zixuan Zhuang, Xuyang Yang, Ziqiang Wang

**Affiliations:** 1 Department of General Surgery, West China Hospital, Sichuan University, Chengdu 610041, China; 2 Colorectal Cancer Center, West China Hospital, Sichuan University, Chengdu 610041, China

**Keywords:** rectal cancer, lateral lymph nodes, bibliometric analysis

## Abstract

Tremendous progress has been made in the field of lateral lymph nodes (LLNs) in rectal cancer, but no bibliometric analysis in this field has been carried out and published. To reveal the current status and trends in LLNs in rectal cancer, this bibliometric analysis was performed. Cooperation network, co-citation and keyword co-occurrence analyses were conducted. Annual publication, cooperation relationships among authors, institutions and countries, co-cited journal, co-cited author, co-cited reference and keywords were the main outcomes. A total of 345 studies were included in this bibliometric analysis. The number of articles published in this field has been increasing year by year. The authors, institutions and countries worked closely together in this field. Japan has the largest number of published articles, accounting for 51.59% of the total publications. *International Journal of Colorectal Disease* (30 papers, 8.70%) published the most papers in this field. The JCOG0212 trial was the most cited article. Preoperative chemoradiotherapy, multicenter, lateral lymph node dissection (LLND) and metastasis are recent hot keywords, and LLND had the highest burst strength. In conclusion, this bibliometric analysis found that Japanese institutions and authors dominated the field of LLNs in rectal cancer. The JCOG0212 trial was the most influential article, which had a significant impact on the development of guidelines. LLND is a hotspot in this field with the highest burst strength. Further prospective studies are needed in this field.

## 1. Introduction

Rectal cancer is one of the leading causes of cancer-related death worldwide [1,2]. With advances in colorectal surgery and adjuvant therapy in recent years, patients’ survival has greatly improved [3,4]. Preoperative neoadjuvant chemoradiotherapy (nCRT) followed by total mesenteric excision (TME) has become a standard mode of treatment for locally advanced rectal cancer (LARC) [5,6]. Nevertheless, how to manage patients with lateral lymph node metastasis (LLNM) is still a tough problem.

There are two main lymphatic drainage pathways in the lower rectum. The major one is through mesorectal nodes, superior rectal nodes to inferior mesenteric artery (IMA) nodes. TME surgery, first proposed by Heald in 1982, has become the standard surgical technique [7], which can completely remove rectal cancer lesions and mesorectal nodes. Another pathway is through lateral lymph nodes (LLNs) [8]. This part of the lymph nodes is beyond the scope of the TME.

Previous studies revealed that the LLNM rate of LARC is approximately 10–25% [9,10,11]. Patients with LLNM have a poor prognosis and a high local recurrence (LR) rate [12]. However, colorectal surgeons have different views on LLNs. In Western countries, nCRT plus TME is recommended for these patients because LLNM is considered a systemic metastatic disease [5,6]. However, in Japan, LLNM is considered a regional disease [9]; therefore, the Japanese Society for Cancer of the Colon and Rectum (JSCCR) guidelines 2019 for the treatment of colorectal cancer recommend that lateral pelvic lymph node dissection (LLND) should be performed for T3/T4 rectal cancer below the peritoneal reflection, regardless of whether LLNM was suspected [13]. Therefore, the field of LLNs in rectal cancer is complex and remains controversial.

Currently, there are still many studies on LLNs in rectal cancer published. However, to the best of our knowledge, no bibliometric analysis in this field has been carried out and published. In this study, we performed a bibliometric analysis to describe the literature related to LLNs in rectal cancer from 1994 (the first article published) to 2022 to reveal the current status and trends in this field. Cooperation networks were constructed to investigate the cooperation relationships among countries, institutions and authors. Co-citation analysis was conducted to investigate the journals, authors and literature that have made outstanding contributions in this field. Keyword co-occurrence analysis was performed to investigate the research focus.

## 2. Materials and Methods

### 2.1. Search Strategy

The WOS core database was used for this bibliometric analysis. This database was searched on 8 August 2022 for all articles related to lateral lymph nodes in rectal cancer from 1955 to 8 August 2022. The detailed search strategy was as follows: TS = (Rectal Neoplasms OR Neoplasm, Rectal OR Cancer, Rectal Neoplasm OR Rectum Neoplasms OR Neoplasm, Rectum OR Rectum Neoplasm OR Rectal Tumors OR Rectal Tumor OR Tumor, Rectal OR Neoplasms, Rectal OR Cancer of Rectum OR Rectum Cancers OR Rectal Cancer OR Cancer, Rectal OR Rectal Cancers OR Rectum Cancer OR Cancer, Rectum OR Cancer of the Rectum) AND TS = (lateral lymph node OR lateral lymph nodes OR lateral pelvic lymph node OR lateral pelvic lymph nodes).

### 2.2. Inclusion Criteria

The inclusion criteria were as follows: the manuscript was based on the theme of lateral lymph nodes in rectal cancer.

### 2.3. Exclusion Criteria

The exclusion criteria were as follows: (1) written in non-English; (2) letters, case reports, editorials and meeting abstracts; (3) study not related to the research topic after browsing the titles and abstracts. Titles and abstracts were independently reviewed by two of our investigators after the literature search. Full articles were retrieved and reviewed if abstracts met the inclusion criteria. Discordant opinions were resolved through consultation with a third investigator or group discussion.

### 2.4. Software and Parameters

Annual publication was analyzed with Endnote X9 and plotted using GraphPad Prism v9.3.1. The top 10 most productive journals were also generated by Endnote X9. Then, bibliometric methods were used to analyze indicators such as author, institution, country, co-cited journal, co-cited author, co-cited reference and keywords with CiteSpace (6.1. R3) software [14]. The parameters of CiteSpace in this study were set as follows: time slicing was chosen from January 1994 to August 2022, year per slice was set to one, node types were selected one at a time, the method to assess the strength of links was chosen as Cosine, scope was set within slices, and top 50 levels of the most cited or occurring items from each slice were selected as the selection criteria.

Each node represented a single author, institution, country, journal, reference or a set of keywords. The node size indicates the frequency of occurrence. In other words, the larger the node is, the higher the frequency of occurrence. The different colors of nodes (from brown to green) from inside to outside indicate the years from 1994 to 2022. Connection lines between nodes represent collaborative, co-occurrence or co-citation relationships. The different colors of connection lines (from brown to green) also indicate the years from 1994 to 2022. In the co-citation analysis, nodes with the outermost purple ring represent that the references have a high betweenness centrality (BC), which is an index that measures the importance of a node [15]. In other words, these references with high BC might play an important bridge role in similar studies.

## 3. Results

A total of 743 publications on lateral lymph nodes in rectal cancer were published on the Web of Science from 1994 to 8 August 2022. Ultimately, 345 studies were included in this bibliometric analysis after excluding 398 papers that did not meet the inclusion eligibility criteria (Figure S1, see the supplementary file).

**Figure 1:**
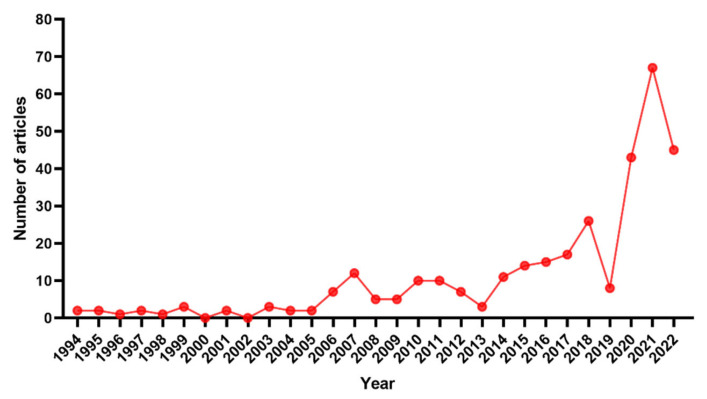
Annual publications on lateral lymph nodes in rectal cancer from 1994 to 2022.

We counted the annual number of publications on lateral lymph nodes in rectal cancer from 1994 to 2022, as shown in Figure 1. This line chart could be divided into three periods: the low-level period (1994–2005), fluctuation rising period (2006–2018) and rapid growth period (2019–2022). Specifically, publications on lateral lymph nodes in rectal cancer began to appear in 1994 and maintained low levels between 1995 and 2005. Then, they increased with fluctuation from 2016 to 2018, followed by a rapid growth period in the last 4 years. As of 8 August, 45 studies about lateral lymph nodes in rectal cancer have been published this year. Overall, the number of articles published in this field has been increasing year by year.

### 3.1. Cooperation Network

**Figure 2:**
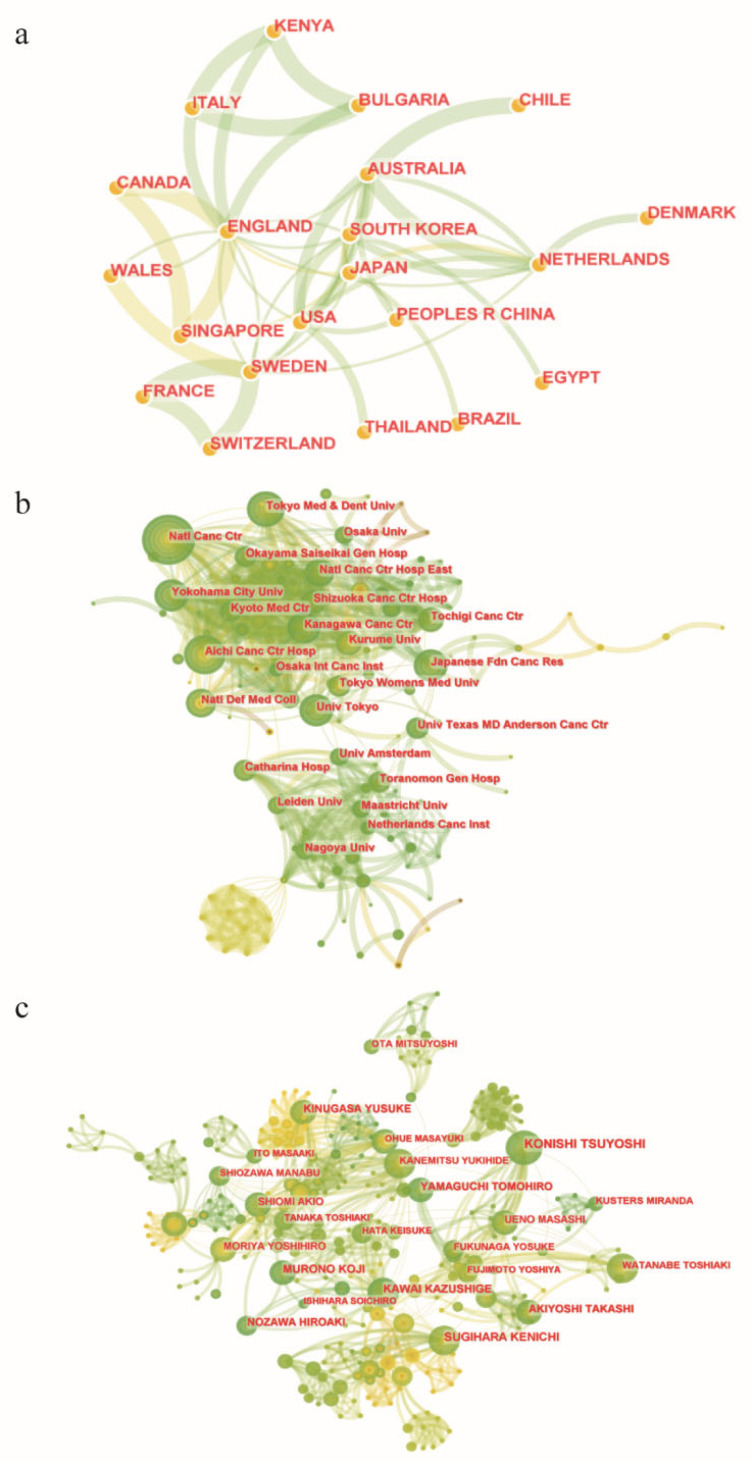
Cooperation networks related to lateral lymph nodes in rectal cancer. (**a**) Cooperation network among countries. (**b**) Cooperation network among institutions. (**c**) Cooperation network among authors. Each node represents a single country institution or author. The node size indicates the frequency of occurrence. Connection lines between nodes represent collaborative relationships. The different colors of nodes/connection lines (from brown to green) from inside to outside indicate the years from 1994 to 2022.

To investigate the cooperation relationships among countries, an international cooperation network was constructed (Figure 2a). It showed that Japan, Republic of Korea, the Netherlands, Australia and England carried out extensive cooperation with other countries. England cooperated with 10 countries, including Bulgaria, Kenya, Italy, Wales, Singapore, Sweden, America, Japan, Republic of Korea and Australia. China mainly cooperated closely with America and Australia. We further calculated the top 10 productive countries related to lateral lymph nodes in rectal cancer (Table 1). Japan has the largest number of published articles, accounting for 51.59% of the total publications. In addition, they published relevant articles as early as 1994. China ranks second (48, 13.91%) in the number of publications.

Then, we performed an institutional cooperation analysis (Figure 2b), which revealed the close relationships among these institutions, including the National Cancer Center of Japan, Tokyo Medical and Dental University and Yokohama City University. The National Cancer Center of Japan, Tokyo University and Tokyo Medical and Dental University were the top three most productive institutions related to lateral lymph nodes in rectal cancer. The top 10 most productive institutions are listed in Table 2. Of note, all other institutions are from Japan except Sichuan University, which is located in China.

Similarly, we further summarized the top 10 productive authors related to lateral lymph nodes in rectal cancer (Table 3) and constructed an author cooperation analysis (Figure 2c). The top 10 productive authors are all from Japan, and Konishi Tsuyoshi is the author with the largest number of published articles (13 papers since 2014).

Overall, authors, institutions and countries worked closely together on the research on lateral lymph nodes in rectal cancer. Japan and Japanese institutions and authors dominated this field regarding the number of publications.

### 3.2. Co-Citation Analysis

To investigate the journals, authors and studies that have made outstanding contributions in the field of lateral lymph nodes in rectal cancer, a co-citation analysis was conducted.

**Figure 3:**
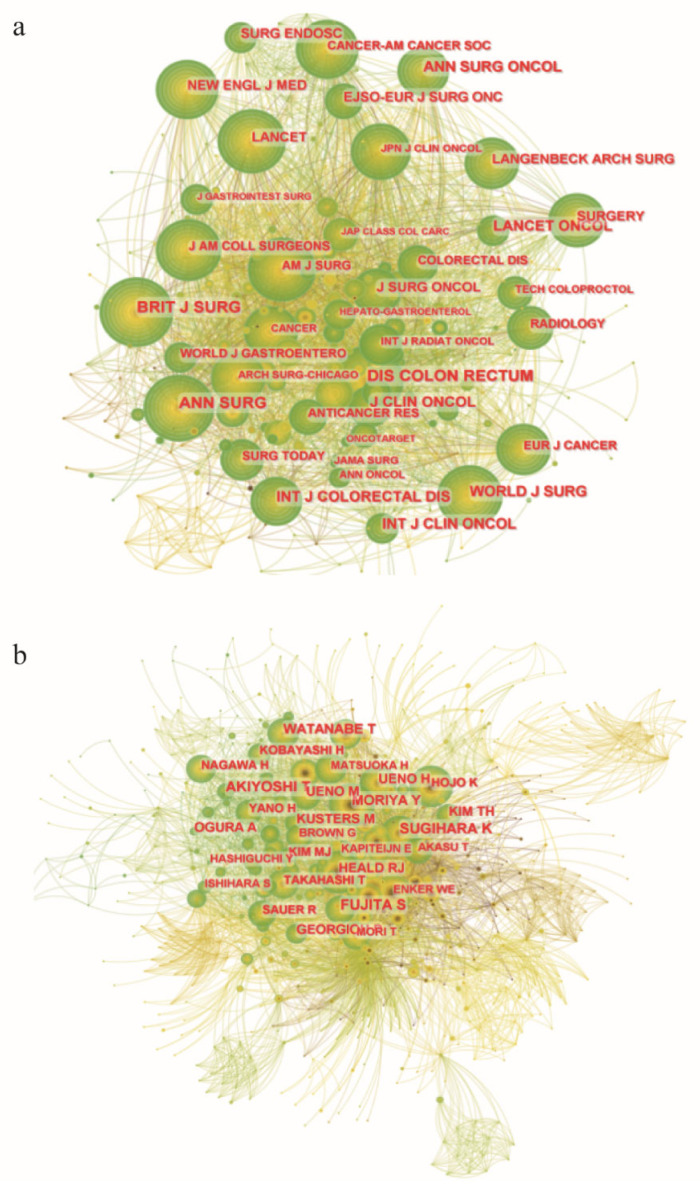
Co-citation networks related to lateral lymph nodes in rectal cancer. (**a**) Co-citation network among journals. (**b**) Co-citation network among authors. Each node represents a single journal or author. The node size indicates the frequency of occurrence. Connection lines between nodes represent co-citation relationships. The different colors of nodes/connection lines (from brown to green) from inside to outside indicate the years from 1994 to 2022.

The top 10 productive journals and top 10 co-cited journals are listed in Table 4 and Table 5. The *International Journal of Colorectal Disease* (30 papers, 8.70%) published the most papers in this field, followed by *Diseases of the Colon and Rectum* (25 papers, 7.25%) and *Annals of Surgical Oncology* (22 papers, 6.38%). In fifth place, *British Journal of Surgery* had the highest Impact Factor (IF) of 11.122. Among the top 10 most productive journals, half were classified as Quartile 1 (Q1) in Journal Citation Report (JCR). Regarding the co-cited journals, *Diseases of the Colon and Rectum* was the most frequently co-cited journal (289 citations), followed by *Annals of Surgery* (272 citations) and *British Journal of Surgery* (261 citations). Among the top 10 co-cited journals, *The New England Journal of Medicine* had the highest IF of 176.079, and seven journals were classified as Q1 in JCR. The network of journals was further constructed (Figure 3a) and revealed that journals with high IFs are commonly co-cited, including *The New England Journal of Medicine*, *Lancet*, etc. When considering both the number of publications and citations, *Diseases of the Colon and Rectum* has made great contributions to the field of lateral lymph nodes in rectal cancer.

Similarly, we summarized the top 10 co-cited authors related to lateral lymph nodes in rectal cancer (Table 6). Shin Fujita (213 citations) was the most frequently co-cited author, followed by Kenichi Sugihara (181 citations) and Takashi Akiyoshi (citations). Of note, the top 5 co-cited authors were all from Japan, and seven of the top 10 co-cited authors also came from Japan. The other three authors were from the Netherlands, the United Kingdom and Republic of Korea, respectively. The co-citation network of authors is shown in Figure 3b.

**Figure 4:**
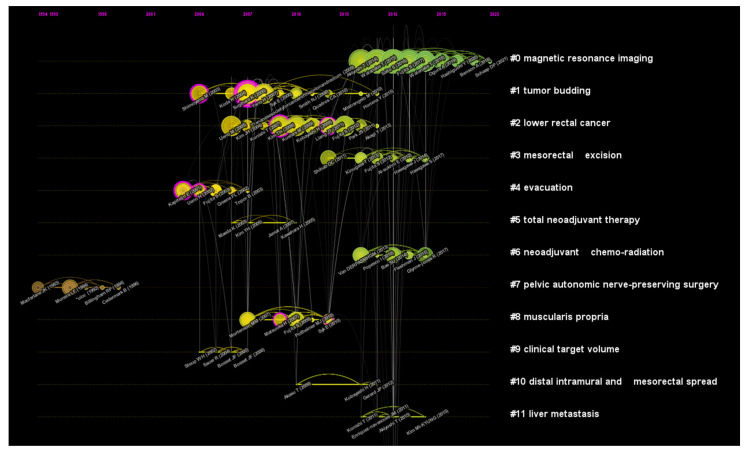
A timeline view for co-cited references related to lateral lymph nodes in rectal cancer. Each node represents a single reference. The node size indicates the frequency of occurrence. Connection lines between nodes represent co-citation relationships. The different colors of nodes/connection lines (from brown to green) from inside to outside indicate the years from 1994 to 2022. Nodes with the outermost purple ring represent that the references have a high betweenness centrality (BC).

To further investigate the core literature that promotes the development of this field, a co-citation analysis of references was conducted. We list the top 10 co-cited references related to lateral lymph nodes in rectal cancer in Table 7. Mesorectal Excision With or Without Lateral Lymph Node Dissection for Clinical Stage II/III Lower Rectal Cancer (JCOG0212): A Multicenter, Randomized Controlled, Noninferiority Trial published in *Annals of Surgery* was the most cited article. The co-citation network of references is shown in Figure 4. It is a timeline graph showing the progress of different research directions in this field over time. Initially, researchers mainly focused on pelvic autonomic nerve-preserving surgery, evacuation and tumor budding. More recently, attention has been given to magnetic resonance imaging, lower rectal cancer, mesorectal excision and neoadjuvant chemo-radiation. The core references are listed in the graph, and nodes with the outermost purple circles had higher BC.

### 3.3. Co-Occurrence Analysis

**Figure 5:**
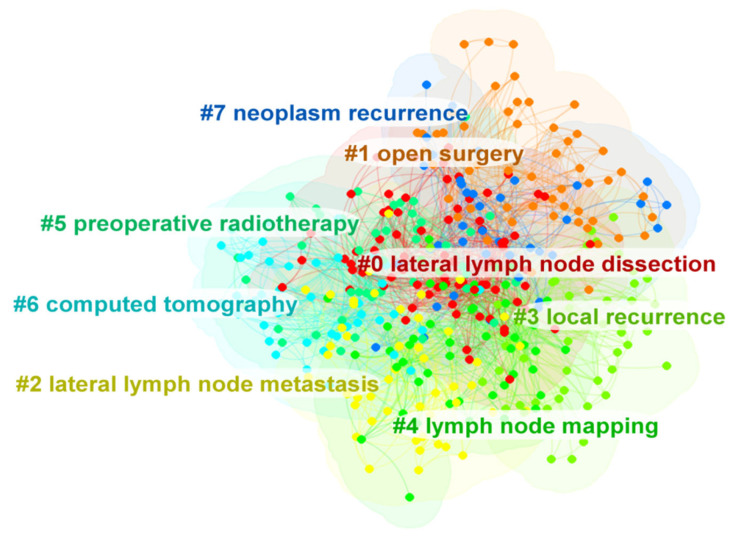
The cluster of keywords related to lateral lymph nodes in rectal cancer. Each point represents a keyword. Different clusters were marked different colors. The lines between the points represent two keywords with co-occurrence relationship.

To investigate the research focus in the field of lateral lymph nodes in rectal cancer, a keyword co-occurrence analysis was performed. The top 10 frequent keywords in this field were rectal cancer, total mesorectal excision, surgery, dissection, carcinoma, preoperative chemoradiotherapy, metastasis, recurrence, resection and lateral lymph node dissection (Table 8). By cluster analysis, all the keywords were mainly assigned to several clusters: lateral lymph node dissection, open surgery, lateral lymph node metastasis, local recurrence, lymph node mapping, preoperative radiotherapy, computed tomography and neoplasm recurrence (Figure 5).

**Figure 6:**
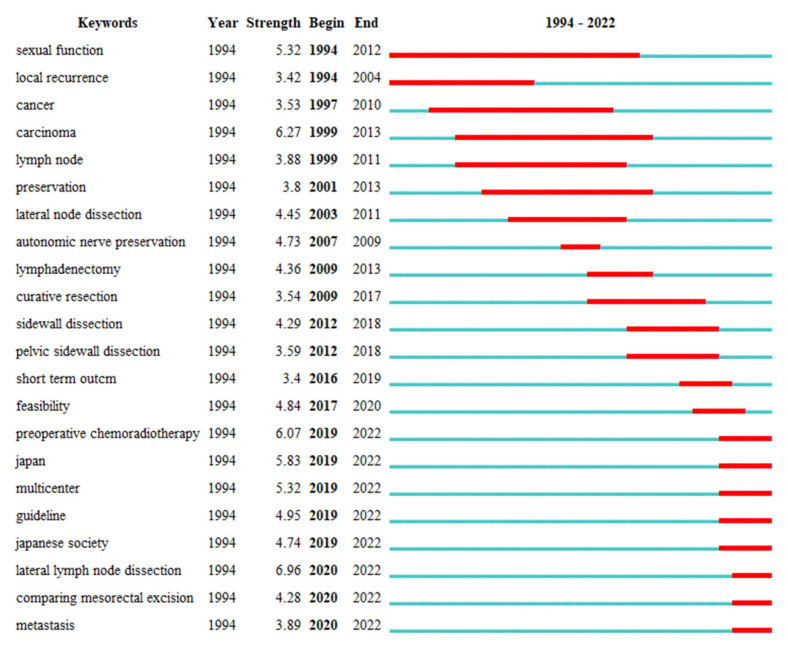
Keywords with burst periods from 1994 to 2022 related to lateral lymph nodes in rectal cancer. Bursts are indicated by red sections on the blue timeline, revealing the start year, the end year and the burst strength.

To identify the frontiers of research in this field, a keyword burst analysis was further conducted. Keyword burst analysis is a kind of analysis in CiteSpace that is used to discover the decline or rise of a keyword in a certain period of time. Some keywords burst from 1994, including sexual function and local recurrence (Figure 6). Several keywords burst around 2010, such as lymphadenectomy, curative resection and pelvic sidewall dissection. In recent years, preoperative chemoradiotherapy, multicenter, lateral lymph node dissection, metastasis, etc., have burst. It is worth mentioning that lateral lymph node dissection had the highest burst strength (6.96), which is and will remain a hot topic in the coming years.

## 4. Discussion

To the best of our knowledge, this is the first bibliometric analysis to describe the literature related to LLNs in rectal cancer. Due to the rapid evolution of research on LLNs in rectal cancer, it may be challenging for researchers and colorectal surgeons to identify the most influential studies and the hot topics in this field. Therefore, we conducted this bibliometric analysis using CiteSpace. Japan has the largest number of published articles. All the top 10 most productive institutions are from Japan except Sichuan University. Konishi Tsuyoshi is the author with the largest number of published articles. The *International Journal of Colorectal Disease* has published the most papers in this field, but *Diseases of the Colon and Rectum* was the most frequently co-cited journal. Shin Fujita was the most frequently co-cited author. The JCOG0212 trial published in *Annals of Surgery* was the most cited article. Preoperative chemoradiotherapy, multicenter, lateral lymph node dissection and metastasis are the recent hot keywords, and LLND had the highest burst strength.

Rectal cancer is one of the most common malignant tumors with high morbidity and mortality [1,2]. Currently, there is still controversy regarding the treatment of LLNs between Western and Eastern colorectal surgeons. In Western countries, nCRT plus TME is recommended to treat patients with clinical stage II/III rectal cancer or suspicious LLNM [5,6]. LLND was not recommended because it causes more postoperative complications and does not provide additional oncological benefits [16,17]. Conversely, Japanese colorectal surgeons tend to consider LLNs as regional lymph nodes, and the latest JSCCR guideline recommended prophylactic LLND for rectal cancer patients [13]. Therefore, many studies related to LLNs or LLND have been conducted in Japan.

Japan has made outstanding contributions to this field. This bibliometric analysis showed that Japan has the largest number of published articles, and 9 of the top 10 most productive institutions were from Japan. Furthermore, all top 10 productive authors and the top 5 co-cited authors were Japanese. Of note, our team (Sichuan University), as the only non-Japanese institution of the top 10 productive institutions, has started focusing on this field [18,19,20,21,22,23]. In summary, although some institutions of other countries began to appear, Japanese institutions and authors dominated the field of LLNs in rectal cancer.

It is difficult to decide who contributed most to this field. Konishi Tsuyoshi was the most productive author, and Shin Fujita was the most frequently co-cited author. When considering both the number of publications and citations, Kenichi Sugihara made a great contribution to this field. However, as the third co-cited author, Takashi Akiyoshi published two highly cited articles, both included in the top 10 co-cited references.

The most influential reference must be the JCOG0212 study [24]. It is a multicenter randomized controlled trial (RCT) in Japan showing that the noninferiority of TME to TME plus LLND could not be confirmed and LLND could significantly reduce LR by 5.2%. This study had a significant impact on the development of guidelines. Although there was no significant difference in overall survival (OS) or relapse-free survival (RFS) between the two groups, patients might benefit from LLND from the perspective of local control. However, this trial only included patients without clinical LLNM, and these patients did not receive nCRT, which limits the generalization of this finding. In addition, our previous meta-analysis, which compared nCRT + TME with nCRT + TME + LLND, also revealed that LLND could significantly reduce local lateral recurrence (LLR) [18]. A recent propensity score matching study showed that clinical LLNM patients had poor OS and DFS, even after LLND, which means that rectal cancer patients who received LLND might gain better long-term survival [25]. However, the decision to perform LLND should be made carefully due to postoperative complications. Therefore, it is important to determine the LLND indications. A previous study revealed that LLNs with a short-axis diameter ≥8 mm before nCRT were an independent risk factor for pathologically positive LLNs [26]. Another study further reported that young age and a short distance from the anal verge were independent risk factors [27]. The Lateral Node Study Consortium of Japan found that a short-axis LLN size of more than 7 mm on primary magnetic resonance imaging (MRI) led to a high LLR [28]. In addition, there was no LLR at 3 years in patients with good shrinkage of LLNs after nCRT (the short axis decreased from more than 7 mm on the primary MRI to less than 4 mm on restaging MRI). However, patients with LLNs whose short axes were still greater than 4 mm on restaging MRI had a high LLR rate (52.3% at 5 years) [29]. Therefore, they recommended restaging MRI when deciding whether to perform LLND and pointed out that LLND might be omitted for the above patients with good shrinkage. Further prospective studies are needed in this field.

Actually, LLND is always a hot topic in this field. Keyword burst analysis, which can reflect research hotspots in academic fields, demonstrated that LLND had the highest burst strength (6.96), starting from 2020. Of note, LLND began to appear in 1994, which means that it has been studied from a very early time and has become a hotspot in recent years. We believe part of the reason for this phenomenon may be the publication of JCOG0212.

Apart from LLND, the cluster analysis showed that open surgery, lateral lymph node metastasis, local recurrence, lymph node mapping, preoperative radiotherapy, computed tomography and neoplasm recurrence were also hot topics in this field. Overall, researchers have mainly focused on which intervention (nCRT or LLND or nCRT + LLND) can reduce the LR of patients with LARC. We also observed that colorectal surgeons have begun to focus on the improvement of LLND surgical techniques [21,30,31,32,33,34]. However, high-quality studies (RCTs) are still lacking.

This study has several limitations. First, this bibliometric analysis only included the WOS core database. Second, non-English studies were excluded. Third, some studies unrelated to the topic were manually removed. All of the abovementioned factors might lead to selection bias.

## 5. Conclusions

This bibliometric analysis found that Japanese institutions and authors dominated the field of LLNs in rectal cancer. The JCOG0212 trial was the most influential article, which had a significant impact on the development of guidelines. LLND is a hotspot in this field with the highest burst strength.

## Figures and Tables

**Table 1:** The top 10 most productive countries related to lateral lymph nodes in rectal cancer.

Rank	Country	Article Counts	Percentage	Year of the First Published Article
1	JAPAN	178	51.59	1994
2	CHINA	48	13.91	2007
3	REPUBLIC OF KOREA	31	8.99	2007
4	USA	24	6.96	2006
5	NETHERLANDS	15	4.35	2010
6	AUSTRALIA	14	4.06	2009
7	ENGLAND	10	2.90	2008
8	SWEDEN	9	2.61	1999
9	BRAZIL	5	1.45	2006
10	SINGAPORE	5	1.45	2010

**Table 2:** The top 10 most productive institutions related to lateral lymph nodes in rectal cancer.

Rank	Institution	Article Counts	Percentage	Year of the First Published Article
1	National Cancer Center of Japan	23	6.67	2003
2	Tokyo University	20	5.80	2012
3	Tokyo Medical and Dental University	17	4.93	2006
4	Yokohama City University	16	4.64	2007
5	Japanese Foundation for Cancer Research	16	4.64	2012
6	National Cancer Center Hospital East	12	3.48	2015
7	Sichuan University	11	3.19	2007
8	Shizuoka Cancer Center Hospital	11	3.19	2015
9	Kanagawa Cancer Center	11	3.19	2007
10	Aichi Cancer Center Hospital	11	3.19	2006

**Table 3:** The top 10 most productive authors related to lateral lymph nodes in rectal cancer.

Rank	Authors	Country	Article Counts	Percentage (n/345)	Year of the First Published Article
1	Konishi Tsuyoshi	Japan	13	3.77	2014
2	Kenichi Sugihara	Japan	11	3.19	2009
3	Murono Koji	Japan	10	2.90	2016
4	Kawai Kazushige	Japan	10	2.90	2016
5	Yamaguchi Tomohiro	Japan	9	2.61	2015
6	Kinugasa Yusuke	Japan	9	2.61	2012
7	Akiyoshi Takashi	Japan	9	2.61	2011
8	Nozawa Hiroaki	Japan	9	2.61	2018
9	Ueno Masashi	Japan	8	2.32	2011
10	Moriya Yoshihiro	Japan	8	2.32	2009

**Table 4:** The top 10 most productive journals related to lateral lymph nodes in rectal cancer.

Rank	Journal	Article Counts	Percentage	IF	Quartile in JCR
1	*International Journal of Colorectal Disease*	30	8.70	2.796	Q2
2	*Diseases of the Colon and Rectum*	25	7.25	4.412	Q1
3	*Annals of Surgical Oncology*	22	6.38	4.339	Q1
4	*Surgical Endoscopy and Other Interventional Techniques*	15	4.35	3.453	Q2
5	*British Journal of Surgery*	15	4.35	11.122	Q1
6	*Anticancer Research*	12	3.48	2.435	Q4
7	*Surgery Today*	11	3.19	2.540	Q2
8	*Langenbeck’s Archives of Surgery*	9	2.61	2.895	Q2
9	*Colorectal Disease*	9	2.61	3.917	Q1
10	*European Journal of Surgical Oncology*	9	2.61	4.037	Q1

JCR, Journal Citation Report.

**Table 5:** The top 10 most co-cited journals related to lateral lymph nodes in rectal cancer.

Rank	Journal	Number of Citations	IF	Quartile in JCR
1	*Diseases of the Colon and Rectum*	289	4.412	Q1
2	*Annals of Surgery*	272	13.787	Q1
3	*British Journal of Surgery*	261	11.122	Q1
4	*Annals of Surgical Oncology*	233	4.339	Q1
5	*Lancet Oncology*	183	54.433	Q1
6	*International Journal of Colorectal Disease*	171	2.796	Q2
7	*International Journal of Clinical Oncology*	170	3.85	Q3
8	*Journal of Clinical Oncology*	168	50.717	Q1
9	*World Journal of Surgery*	160	3.282	Q2
10	*The New England Journal of Medicine*	137	176.079	Q1

JCR, Journal Citation Report.

**Table 6:** The top 10 most co-cited authors related to lateral lymph nodes in rectal cancer.

Rank	Authors	Country	Number of Citations
1	Shin Fujita	Japan	213
2	Kenichi Sugihara	Japan	181
3	Takashi Akiyoshi	Japan	177
4	Toshiaki Watanabe	Japan	153
5	Yasuhiro Moriya	Japan	135
6	Miranda Kusters	the Netherlands	123
7	Richard J Heald	the United Kingdom	115
8	Tae Hyun Kim	Republic of Korea	114
9	Atsushi Ogura	Japan	109
10	Masashi Ueno	Japan	106

**Table 7:** The top 10 co-cited references related to lateral lymph nodes in rectal cancer.

Rank	Title	Journal	First Author	Number of Citations	Publication Time
1	Mesorectal Excision With or Without Lateral Lymph Node Dissection for Clinical Stage II/III Lower Rectal Cancer (JCOG0212): A Multicenter, Randomized Controlled, Noninferiority Trial	*Annals of surgery*	Shin Fujita	117	2017
2	Neoadjuvant (Chemo)radiotherapy With Total Mesorectal Excision Only Is Not Sufficient to Prevent Lateral Local Recurrence in Enlarged Nodes: Results of the Multicenter Lateral Node Study of Patients With Low cT3/4 Rectal Cancer	*Journal of clinical oncology*	Atsushi Ogura	85	2019
3	Japanese Society for Cancer of the Colon and Rectum (JSCCR) guidelines 2016 for the treatment of colorectal cancer	*International journal of colorectal disease*	Toshiaki Watanabe	51	2018
4	Japanese Society for Cancer of the Colon and Rectum (JSCCR) guidelines 2019 for the treatment of colorectal cancer	*International journal of colorectal disease*	Yojiro Hashiguchi	50	2020
5	Oncological Outcomes of Lateral Pelvic Lymph Node Metastasis in Rectal Cancer Treated With Preoperative Chemoradiotherapy	*Diseases of the colon and rectum*	Soichiro Ishihara	44	2017
6	Indications for Lateral Pelvic Lymph Node Dissection Based on Magnetic Resonance Imaging Before and After Preoperative Chemoradiotherapy in Patients with Advanced Low-Rectal Cancer	*Annals of surgical oncology*	Takashi Akiyoshi	40	2015
7	Selective lateral pelvic lymph node dissection in patients with advanced low rectal cancer treated with preoperative chemoradiotherapy based on pretreatment imaging	*Annals of surgical oncology*	Takashi Akiyoshi	39	2014
8	Japanese Society for Cancer of the Colon and Rectum (JSCCR) Guidelines 2014 for treatment of colorectal cancer	*International journal of colorectal disease*	Toshiaki Watanabe	35	2015
9	Male sexual dysfunction after rectal cancer surgery: Results of a randomized trial comparing mesorectal excision with and without lateral lymph node dissection for patients with lower rectal cancer: Japan Clinical Oncology Group Study JCOG0212	*European journal of surgical oncology*	S Saito	35	2016
10	Lateral Nodal Features on Restaging Magnetic Resonance Imaging Associated With Lateral Local Recurrence in Low Rectal Cancer After Neoadjuvant Chemoradiotherapy or Radiotherapy	*Jama surgery*	Atsushi Ogura	35	2019

**Table 8:** The top 10 frequent keywords related to lateral lymph nodes in rectal cancer.

Rank	Keyword	Article Counts	Percentage	Year of the First Published Article
1	Rectal cancer	182	52.75	1994
2	Total mesorectal excision	132	38.26	1996
3	Surgery	99	28.70	1999
4	Dissection	96	27.83	2003
5	Carcinoma	77	22.32	1995
6	Preoperative chemoradiotherapy	76	22.03	2008
7	Metastasis	75	21.74	2007
8	Recurrence	69	20.00	1999
9	Resection	67	19.42	1995
10	Lateral lymph node dissection	66	19.13	2004

## Data Availability

The original data are available from the corresponding author upon request.

## Supplementary Materials

The following supporting information can be downloaded at: https://www.mdpi.com/article/10.3390/healthcare11101362/s1, Figure S1: Flowchart of the selection process.
