# Supplementary figures and images for: Global Status of Research on Lateral Lymph Nodes in Rectal Cancer from 1994 to 2022: A Bibliometric Analysis
Source: Healthcare (Basel). 2023 May 9;11(10):1362. doi: 10.3390/healthcare11101362 (PMC10218116; doi:10.3390/healthcare11101362)

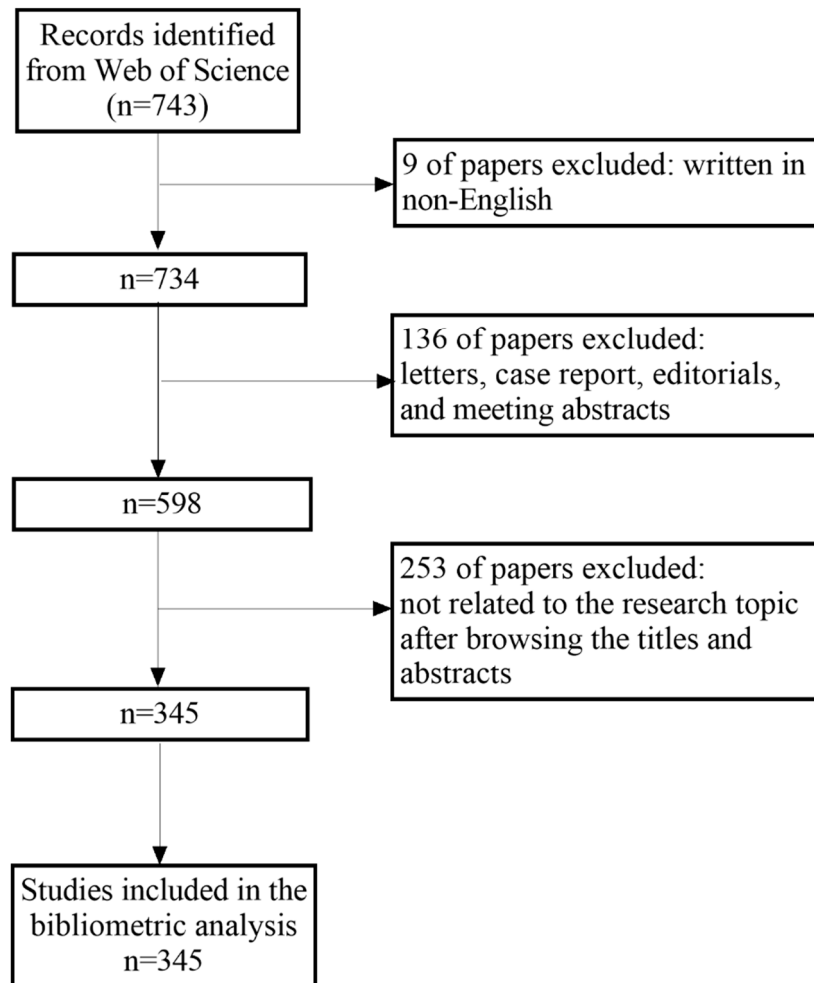

Figure S1. Flowchart of the selection process.

Supplement: Supplementary file 1 [file healthcare-11-01362-s001.zip › healthcare-2335375-supplementary.pdf]
